# Lumpy skin disease in cattle: Frequency of occurrence in a dairy farm and a preliminary assessment of its possible impact on Egyptian buffaloes

**DOI:** 10.4102/ojvr.v84i1.1393

**Published:** 2017-03-28

**Authors:** Mahmoud M. Elhaig, Abdelfattah Selim, Mohamed Mahmoud

**Affiliations:** 1 Department of Animal Medicine (Infectious Diseases), Suez Canal University, Egypt; 2 Department of Animal Medicine (Infectious Diseases), Benha University, Egypt

## Abstract

Lumpy skin disease (LSD) is an endemic infectious disease of cattle in Egypt. This survey aimed to define the prevalence of clinical and sub-clinical LSD virus (LSDV) infection among cattle and investigate their contact with water buffaloes (*Bubalus bubalis*) in order to improve the understanding of LSD epidemiology. Cattle and buffalo were examined owing to the appearance of skin lesions. Because clinical signs were consistent with LSDV infection, samples from cattle in a non-grazing dairy farm (*n* = 450) were submitted for LSDV testing together with those from the in-contact buffaloes (*n* = 100). Results revealed that the intra-herd percentage of cattle infected with LSDV varied with the detection method. This ranged from 22.4% to 65.4% by virus isolation (VI) and polymerase chain reaction (PCR), respectively, in clinical cattle samples, compared to 0% and 10% by VI and PCR in non-clinical cases. Using the neutralising index (NI), LSDV antibodies were found in 100% (*n* = 100) of the tested cow’s sera (NI = > 2.0 and ≥ 3.0), whereas buffalo’s sera (*n* = 34) displayed little increase in antibody level (NI ≥ 1.5). None of the buffalo were positive for LSDV by VI and PCR. In addition, there were no significant differences in LSD prevalence among the cattle with regard to age and sex. In conclusion, the occurrence of LSD in cattle warrants a further epidemiological study of the spread of the disease in the area and adoption of control and prevention strategies. In addition, the PCR assay was confirmed to be useful in the diagnosis of LSDV and for wider epidemiological studies.

## Introduction

Lumpy skin disease (LSD) is a serious viral disease of cattle and is suspected to be transmitted mechanically by blood-feeding arthropods (Magori-Cohen et al. 2012). The disease is caused by the LSD virus (LSDV), which belongs to the *Capripoxvirus* genus of the Poxviridae family (Babiuk et al. 2008). It is endemic in many African countries (Tuppurainen et al. 2011). LSD continues to circulate through the Middle East region and is a grave threat to the rest of Asia and Europe (Abutarbush et al. 2013; Tageldin et al. 2014).

LSDV infection shows large variations in clinical presentation that range from sub-clinical infection to death (Carn & Kitching 1995). These can include fever; eruption of skin nodules covering the neck, back, perineum, tail, hind legs and genital organs; superficial lymph node enlargement and, in a few animals, oedema of the limbs and brisket together with lameness. There are severe economic losses due to emaciation, decreased or cessation of milk production, low weight gain, abortion, myiasis and permanent damage of hides which causes lowering of their commercial value (Abera et al. 2015b; Abutarbush et al. 2013; Al-Salihi 2014). Morbidity and mortality vary greatly depending on the activity of insects, susceptibility and the immune status of cattle. Morbidity ranging from 2% to 85% and even higher has been recorded. Mortality is, however, low (1% – 5%) but can be as high as 40% in some cases (Davies 1991).

LSDV has a limited host range and does not complete its replication cycle in non-ruminant hosts (Shen et al. 2011). Cattle breeds of both sexes and all ages are susceptible to LSDV, but there is some evidence to support that young animals may be more susceptible to the severe form of the disease (Al-Salihi 2014). Moreover, LSD has not been reported in sheep and goats even when kept in a close contact with infected cattle (Davies 1991).

Infection of water buffalo with LSDV under field condition is a controversial matter. Isolation of LSDV from skin lesions of buffalo in Egypt has been described (El-Nahas et al. 2011; Sharawi & El-Rahim 2014), but other workers (Davies 1991) reported that African buffalo (*Syncerus caffer*) and Asian water buffalo (*Bubalus bubalis*) do not show lesions in the field during epizootics of LSD. They do, however, seroconvert.

Transmission of the LSDV has been associated with haematophagous insects, such as mosquitoes and stable flies (Carn & Kitching 1995; Lubinga et al. 2014). Recently, Ixodid ticks are suspected to have a role in the transmission of LSDV in cattle (Tuppurainen et al. 2011). Direct contact between cattle or contact through milking procedure were also reported as potential transmission modes (Magori-Cohen et al. 2012).

This study aimed to demonstrate LSDV infection in cattle using different diagnostic techniques and analyse the risk factors associated with age and sex.

## Materials and methods

### Study area and sample collection

LSD was suspected in a dairy herd from January 2014 to mid-2015 after the appearance of clinical signs suggestive of the disease in cattle in Sharqia Governorate, Egypt. The skin lesions were observed in a non-grazing dairy farm which included both cows (*n* = 450) and buffaloes (*n* = 100). Cattle and buffalo were, however, separated from each other by fences and did not share water or feed troughs. On 17 July 2014, five buffaloes displayed skin lesions. The precise date of onset of clinical signs in the buffaloes was not documented. However, after interviewing the veterinarian and the owner, it was understood that these buffalo lesions were not apparent before the onset of skin lesions that were suggestive of LSD in cows. The cattle and buffalo ages ranged from 6 months up to 1 year, to more than 5 years. The cattle had a history of vaccination with sheep pox vaccine (10^3^ TCID_50_ sheep poxvirus per dose, Veterinary Serum and Vaccine Research Institute [VSVRI], Egypt) since 6 months previously, whereas the buffaloes were never vaccinated. Blood samples and skin biopsies were collected from the 78 cattle and the 5 buffaloes that showed clinical signs. Twenty blood samples were collected from the clinically asymptomatic in-contact cows and buffaloes into Ethylenediaminetetraacetic acid (EDTA) tubes by jugular venepuncture. Samples were transported to the lab on ice with minimal delay for virus detection. Serum samples (*n* = 100) were taken randomly from cattle and the same number of buffalo.

### Virus isolation

Blood and skin biopsies were used for isolation of LSDV according to Tuppurainen, Venter and Coetzer (2005). Briefly, for blood samples, 0.5 mL was inoculated into confluent monolayer MDBK grown in 12-well plates containing the growth medium (Minimum Essential Medium [MEM] with L-glutamine, 0.2% sodium bicarbonate, 5% foetal calf serum and gentamycin 0.05 mg/mL). Twenty-four hours later, the medium was removed and the cells were washed twice with phosphate-buffered saline (PBS) and gentamycin (0.05 mg/mL). The medium was replaced with MEM containing 5% foetal calf serum, l-glutamine, 0.2% sodium bicarbonate and gentamycin (0.05 mg/mL), whereas in case of skin biopsies, tissues were minced and ground with a pestle in a sterile mortar in aid of sterile sand. A suspension of 10 mL of PBS containing antibiotics (for each mL, 0.1 mg gentamycin, 0.05 mg ampicillin, 5 μg amphotericin B) was added, then left to stand overnight at 4 °C. The suspension was centrifuged for 5 min at 2000 rpm to remove any gross particles, and then 0.5 mL of supernatant was transferred into monolayer MDBK cells growing in the medium described above. Cultures were observed daily for the cytopathic effect for 14 days. Further, a passage for another 14 days was required in case of negative culture into fresh monolayers.

### Neutralisation index test

The neutralisation index (NI) test using the serum at a final 1:10 dilution was tested using 10 000 MDBK cells/microtiter well. Neutralisation indices ≥ 1.5 were considered positive for LSDV antibodies. The NI was performed as described by Cottral (1978).

### DNA extraction and polymerase chain reaction amplification

Genomic DNA was extracted from the whole blood and skin biopsies using a High Pure polymerase chain reaction (PCR) Template Preparation Kit (Roche, Germany) following the manufacturer’s instructions. Briefly, 200 µL blood was mixed with 200 µL binding buffer and 40 µL proteinase K and the mixture incubated for 10 min at 70 °C. Isopropanol at 100 µL was then added and mixed well. The DNA samples (200 µL) were extracted following the loading of the mixture in the high purity filter tubes of the kit, and aliquots were stored at -20 °C until used. PCR was performed at a final volume of 25 µL containing 12.5 μL of Go Taq^®^ Green Master Mix 2X (Promega Co., USA), 20 pmol (1.0 μL) of each primer, 3 μL of the DNA extract and DD H_2_O up to 25 µL. For PCR amplification of the 1237-bp fragment of LSDV, using #LSD43U-5’GTGGAAGCCAATTAAGTAGA3’ and LSD1262L-5’TAAGAGGGACATTAGTTCT3’ primers (Stram et al. 2008), amplification was performed as an initial cycle at 95 °C for 1 min, and then 35 cycles of 94 °C for 30 s, 58 °C for 30 s and 72 °C for 70 s and a final extension step of 72 °C for 5 min. In each PCR run, a non-template control was included to detect possible external DNA contamination, and control positive (reference Ismailia LSDV, VSVRI, Abbasia, Egypt) was used for confirmation. Ten microlitres of each amplified product was analysed by agarose gel electrophoresis using a 1-kbp DNA ladder on 1.5% agarose (Roko, Spain), containing 1 μg/mL ethidium bromide in Tris/borate/EDTA buffer and photographed under UV light.

### Statistical analysis

Chi-squared tests were performed (http://vassarstats.net/) to evaluate statistical differences in LSD prevalence in cattle across different sex and age classes (Table 1), whereas the kappa values were calculated to evaluate the agreement between the VI and PCR (http://vassarstats.net/kappa.html).

**TABLE 1:** Prevalence of lumpy skin disease in cattle according to age and sex.

Risk factors	Prevalence rate		*p*

	*N*	%	
**Age**			
< 1 year (*n* = 85)	10	11.8	0.1106
1–3 years (*n* = 200)	32	16.0	-
> 3 years (*n* = 165)	36	21.8	-
**Sex**			
Male (*n* = 190)	31	16.3	0.6290
Female (*n* = 260)	47	18.0	-

**Total (*N* = 450)**	**78**	**17.3**	**-**

The result is statistically significant at *p* < 0.05

## Results

### Case description

Starting from January 2014, the whole herd was examined daily to detect animals that showed typical LSD clinical signs. Suspected cattle (17.3%, 78/450) appeared with small-sized nodules over the trunk and the neck and sometimes accompanied by fever (39.5 °C – 40.0 °C), slight lymphadenopathy and leg oedema. On the contrary, buffaloes (*n* = 5) were found with skin lesions on the hind limbs in the form of hard painless nodules with transient fever for a few days and noticeable swelling in the legs and under the belly while the rest of the buffaloes were non-clinical.

### Prevalence of lumpy skin disease in cattle according to age and sex

As shown in Table 1, in total, 450 cattle were examined clinically. The intra-herd clinical prevalence of LSD in cattle in this study was 17.3% (78/450 heads of the animals). Analysis of the age prevalence of LSD revealed that the highest prevalence was observed in ages of  > 3 years (21.8%, *n* = 36). This was followed by 1–3 years (16%, *n* = 32) and < 1 year (11.8%, *n* = 10) with no statistically significant differences. The prevalence was 18% and 16.3% in females and males, respectively. The difference was not statistically significant between the two sexes.

### Detection of lumpy skin disease virus using virus isolation and polymerase chain reaction assays in cattle and buffalo

Of the 176 representative clinical samples (skin biopsies and blood samples) from cattle, 19.9% and 59.1% were positive on VI and PCR, respectively (Table 2). As tabulated in Table 2, the 78 suspect clinical cases were checked by VI and PCR assays, where 32.1% (skin biopsies) and 12.8% (blood), and 89.7% (skin biopsies) and 40.1% (blood) tested positive by VI and PCR, respectively. Of the 20 blood samples of non-clinical cows, 2 (10%) were positive by PCR assay only. All samples positive by VI were also positive by PCR assay. None of the buffalo samples was positive for LSDV by either assay. Kappa testing showed fair agreement between VI and PCR (kappa = 0.29, 95% confidence interval = 0.0.21–0.39) (Table 3).

**TABLE 2:** Detection of lumpy skin disease virus in blood samples and skin biopsies of cattle and buffaloes.

Cattle samples	Number of samples	VI†		PCR‡	
	
		Positive	%	Positive	%
**Clinical suspect**					
Skin biopsy	78	25	32.1	70	89.7
Blood	78	10	12.8	32	40.1
**Total**	**156**	**35**	**22.4**	**102**	**65.3**
**Non-clinical suspect**					
Blood	20	0	0.0	2	10.0
**Total**	**176**	**35**	**19.9**	**104**	**59.1**

VI, virus isolation; PCR, polymerase chain reaction.

† , None of buffalo’s blood or skin biopsies was positive for lumpy skin disease virus by virus isolation; ‡, none of buffalo’s blood or skin biopsies was positive for lumpy skin disease virus by polymerase chain reaction.

**TABLE 3:** Comparison of viral isolation and the polymerase chain reaction assay targeting lumpy skin disease virus detection.

Test	VI		Total

	Positive	Negative	
**PCR**			
Positive	35	69	104
Negative	0	72	72

**Total**	**35**	**141**	**176**

A kappa value < 0.0 is considered a poor agreement, 0.21–0.40 fair agreement, 0.41–0.60 moderate agreement, 0.61–0.80 substantial agreement and 0.81–1.00 good agreement.

PCR, polymerase chain reaction; VI, virus isolation.

### Seroconversion

**FIGURE 1:**
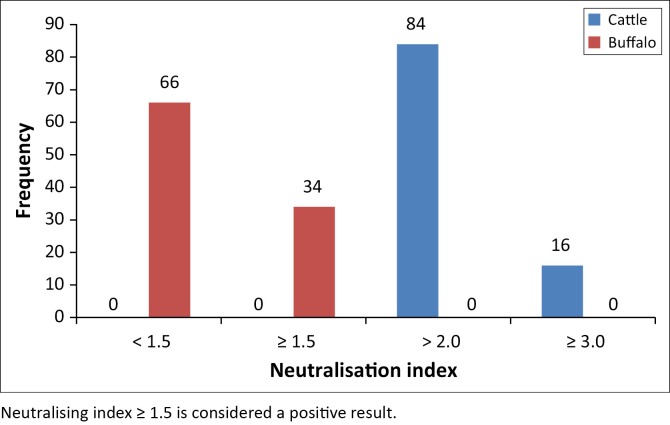
Results of neutralisation index for lumpy skin disease virus from 100 cattle and 100 buffalo.

As shown in Figure 1, seroconversion of LSDV was assessed in 100 sera obtained from cattle by serum neutralisation index. A total of 84 and 16 sera tested positive, with a serum NI > 2.0 and ≥ 3.0, respectively. On the contrary, in buffalo sera, there were 66 sera, including the five buffaloes with skin lesions, negative for LSDV antibody (NI = 0.8–1.5), whereas 34 sera samples displayed a small increase in serum antibody levels (NI ≥ 1.5).

## Discussion

LSD is endemic in Africa, and cattle are the natural hosts of the disease (Tuppurainen et al. 2015). In 1988, LSDV was reported for the first time from cattle in Egypt, whereas water buffalo and sheep which were in direct contact with the affected cattle remained clinically normal (House et al. 1990). Thereafter, LSD reappeared in Egyptian cattle in the summer of 1989 with 2% morbidity (Davies 1991). In 2006, a new and extensive LSD outbreak struck Egypt affecting 16 provinces (Awadin et al. 2011), and the disease re-emerged in 2011 and again in 2014 (Amin, ElMashad & El-Nahas 2015; El-Tholoth & El-Kenawy 2016). The LSD is currently spreading aggressively in the Near East (Tuppurainen et al. 2015). Taking into consideration previous reports and the detection of LSD cases among cattle in this study, it is clear that this economically devastating disease continues to expand its territorial cover in Egypt and is a threat to Eastern Europe, the Balkan countries and parts of Russia and Asia.

The epidemiological data concerning LSDV in Egypt and North Africa are insufficient. In this study, LSD clinically manifested itself by causing less severe clinical signs in cattle in comparison with previous outbreak in 2006. Although annual mass vaccination by the Egyptian authority with sheep pox vaccine (VSVRI, Egypt) has been done, the intra-herd prevalence rate of clinical cases in this study was 17.3% and mostly in adult animals. This could be explained by the fact that not all vaccinated animals develop a protective level of immunity against LSDV (Tuppurainen & Oura 2012), as attested by 11.1% of vaccinated animals that developed LSD in Israel (Stram et al. 2008).

Although prevalence was higher in adult animals when compared to young calves, there was no statistically significant association between animal age and prevalence rate in this study. This finding indicated that young and adult animals are equally exposed to risk. Earlier, Abera et al. (2015b) reported no association among age groups despite higher prevalence in the adult age group.

The low prevalence in calves in this study may be associated with lower susceptibility of calves to biting flies and keeping them where there is less insect activity (Rweyemamu et al. 2000; Troyo et al. 2008). Another possibility may be the presence of passive maternal immunity which protects calves for about 6 months (Weiss 1968). On the contrary, under field conditions, very young calves and lactating and malnourished animals developed severe disease which might have been due to impaired cellular immunity or lack of vaccination (Hunter & Wallace 2001).

There were no significant differences between sex and LSD prevalence in this study despite the slightly higher prevalence of LSD infection in females (18%) and males (16.3%). This finding is similar to that reported by some workers (Ayelet et al. 2014; Tuppurainen & Oura 2012), yet disagrees with findings of others (Abera et al. 2015a; Gari et al. 2010) who indicated that males exhibited a higher disease susceptibility than females because of exposure to stress factors such as fatigue from heavy work. Further, the intra-herd prevalence of the studied non-grazing farm reached 17.3%, which may be related to the presence of large numbers of susceptible animals, poor biosecurity practice, high densities of biting insects around the feedlot areas and improper management of farm waste (Alemayehu et al. 2015). In addition, recently LSD was reported in a dairy cattle farm in Oman where it was possible that the causes of intra-herd spread included improper insect control and inefficient vaccination (Kumar 2011).

In this study, laboratory analyses showed that 19.9% and 59.1% of tested clinical samples from cattle were positive for LSDV using VI and PCR, respectively. None of the buffalo samples was positive for LSDV using either assay. The decreased proportion of positive samples by VI compared to PCR is in agreement with Awad et al. (2010). The low detection rate of VI in this study and previous studies may be attributable to the slow growth of capripoxviruses in cell culture, low virus titres in samples, the need for several passages, small sample sizes or incorrect sample storage and processing (Awad et al. 2010; Bowden et al. 2008; Tuppurainen et al. 2005).

The study indicated a fair agreement between results of the VI and PCR, although virus could not be isolated from all PCR-positive samples (*n* = 69). These results support those of other workers (Awad et al. 2010; Tuppurainen et al. 2005), who reported that the PCR was more sensitive and accurate. The large proportion of positive samples in PCR tests may be related to the assay’s ability to detect LSDV DNA in blood and skin biopsies for a longer period than VI (Tuppurainen et al. 2005). Further, the PCR can detect low genome copies of the pathogen even in the dead state, its rapidity and relative ease of analysis and interpretation (Al-Salihi 2014; Awad et al. 2010).

The role of water buffalo in the epidemiology of LSD remains unclear in Egypt. Evidence of natural infection with LSDV in buffalo has been reported in Egypt (El-Tholoth & El-Kenawy 2016; Sharawi & El-Rahim 2014). In this study, serum samples were collected from cattle and buffalo, and the LSDV intra-herd antibody prevalence was estimated using the NI method. A higher prevalence in cattle was reported by House et al. (1990). The findings also indicate that NI is a valuable diagnostic tool for LSD seroprevalence studies (OIE 2010).

In a previous study, Fagbo et al. (2014) indicated that 5 of 66 buffaloes tested positive for LSDV antibodies with low titres using a serum neutralisation test. In another study (Barnard 1997), no LSDV antibodies were detected in 15 buffaloes from South Africa. Taking into consideration the failure to detect LSDV in buffaloes using VI and PCR and the failure to detect antibody in most cases except in some with poor seroconversion, suggests that the Egyptian water buffalo is not susceptible to LSDV and may serve as an accidental non-adapted host. On the contrary, the low proportion of positive buffalo sera could be false due to unknown specificity of the test and is supported by the fact that the skin nodules were negative for LSDV.

House et al. (1990) reported that only cattle developed LSD, whereas sheep, goats and water buffalo appeared clinically healthy during the LSD outbreak in Ismailia in 1988. Moreover, LSD has been recorded only in cattle at Ismailia abattoir, Egypt, and was never recorded in buffaloes and cattle calves (Ahmed & Dessouki 2013).

Determining the true exposure status of naturally infected animals is often difficult because of several factors affecting the performance of serological tests for capripoxviruses such as past infection and vaccination history (Fagbo et al. 2014). Recently, molecular diagnosis of LSDV was documented from naturally infected water buffalo in Egypt (El-Nahas et al. 2011; Sharawi & El-Rahim 2014). However, the PCR assays used in the studies were not able to distinguish between different capripoxviruses. This conclusion is supported by an earlier study (Greta et al. 1992) which reported a capripox disease in Arabian oryx. It is thought that the cause of infection was because of related capripoxviruses rather than LSDV. Due to the small number of buffaloes studied in this study, the potential role of this species in the epidemiology of LSD in Egypt cannot be confirmed and further large-scale molecular-based surveillance is required to determine the susceptibility of buffalo to LSDV infection.

## Conclusion

LSD is an important disease in cattle, which is still the main natural host, even if the observed clinical form in this study was less severe. Egyptian buffaloes are likely to be more resistant. Nevertheless, in light of evidence for the presence of capripoxviruses having been found in water buffaloes, their potential role in the epidemiology of LSD may warrant further studies.

## References

[CIT0001] Abera Z., Degefu H., Gari G. & Ayana Z, 2015a, ‘Review on epidemiology and economic importance of lumpy skin disease’, *International Journal of Basic and Applied Virology* 4, 08–21.

[CIT0002] Abera Z., Degefu H., Gari G. & Kidane M, 2015b, ‘Sero-prevalence of lumpy skin disease in selected districts of West Wollega zone, Ethiopia’, *BMC Veterinary Research* 11, 135 https://doi.org/10.1186/s12917-015-0432-7 26082259 10.1186/s12917-015-0432-7 PMC4468805

[CIT0003] Abutarbush S., Ababneh M., Al Zoubi I., Al Sheyab O., Al Zoubi M., Alekish M. et al., 2013, ‘Lumpy skin disease in Jordan: Disease emergence, clinical signs, complications and preliminary-associated economic losses’, *Transboundary and Emerging Diseases* 62, 549–554. https://doi.org/10.1111/tbed.12177 24148185 10.1111/tbed.12177

[CIT0004] Ahmed A.M. & Dessouki A.A, 2013, ‘Abattoir-based survey and histopathological findings of lumpy skin disease in cattle at Ismailia abattoir’, *International Journal of Bioscience, Biochemistry and Bioinformatics* 3, 372.

[CIT0005] Alemayehu G., Leta S., Eshetu E. & Mandefro A, 2015, ‘Incidence of lumpy skin disease and associated risk factors among export-oriented cattle feedlots at Adama District, Central Ethiopia’, *Journal of Veterinary Medicine and Animal Health* 7, 128–134. https://doi.org/10.5897/JVMAH2014.0357

[CIT0006] Al-Salihi K, 2014, ‘Lumpy skin disease: Review of literature’, *Mirror of Research in Veterinary Sciences and Animals* 3, 6–23.

[CIT0007] Amin A.A., ElMashad A. & El-Nahas E, 2015, ‘Pathological and virological studies on an outbreak of lumpy skin disease among cattle in Kalubia Governorate-Egypt’, *Journal of Advanced Veterinary Research* 5, 165–175.

[CIT0008] Awad W.S., Ibrahim A.K., Mahran K., Fararh K.M. & Moniem M.I.A, 2010, ‘Evaluation of different diagnostic methods for diagnosis of lumpy skin disease in cows’, *Tropical Animal Health and Production* 42, 777–783. https://doi.org/10.1007/s11250-009-9486-5 19882228 10.1007/s11250-009-9486-5

[CIT0009] Awadin W., Hussein H., Elseady Y., Babiuk S. & Furuoka H, 2011, ‘Detection of lumpy skin disease virus antigen and genomic DNA in formalin-fixed paraffin-embedded tissues from an Egyptian outbreak in 2006’, *Transboundary and Emerging Diseases* 58, 451–457. https://doi.org/10.1111/j.1865-1682.2011.01238.x 21699673 10.1111/j.1865-1682.2011.01238.x

[CIT0010] Ayelet G., Haftu R., Jemberie S., Belay A., Gelaye E., Sibhat B. et al., 2014, ‘Lumpy skin disease in cattle in central Ethiopia: Outbreak investigation and isolation and molecular detection of the virus’, *Revue scientifique et technique (International Office of Epizootics)* 33, 877–887. https://doi.org/10.20506/rst.33.3.2325 25812211 10.20506/rst.33.3.2325

[CIT0011] Babiuk S., Bowden T., Boyle D., Wallace D.B. & Kitching R, 2008, ‘Capripoxviruses: An emerging worldwide threat to sheep, goats and cattle’, *Transboundary and Emerging Diseases* 55, 263–272. https://doi.org/10.1111/j.1865-1682.2008.01043.x 18774991 10.1111/j.1865-1682.2008.01043.x

[CIT0012] Barnard B, 1997, ‘Antibodies against some viruses of domestic animals in southern African wild animals’, *The Onderstepoort Journal of Veterinary Research* 64, 95–110. 9352558

[CIT0013] Bowden T.R., Babiuk S.L., Parkyn G.R., Copps J.S. & Boyle D.B, 2008, ‘Capripoxvirus tissue tropism and shedding: A quantitative study in experimentally infected sheep and goats’, *Virology* 371, 380–393. https://doi.org/10.1016/j.virol.2007.10.002 17988703 10.1016/j.virol.2007.10.002 PMC9955785

[CIT0014] Carn V. & Kitching R, 1995, ‘The clinical response of cattle experimentally infected with lumpy skin disease (Neethling) virus’, *Archives of Virology* 140, 503–513. https://doi.org/10.1007/BF01718427 7733823 10.1007/BF01718427

[CIT0015] Cottral G.E, 1978, *Manual of standardized methods for veterinary microbiology*, Cornell University Press, Ithaca, NY.

[CIT0016] Davies F, 1991, ‘Lumpy skin disease of cattle: A growing problem in Africa and the Near East’, *World Animal Review* 68, 37–42.

[CIT0017] El-Nahas E., El-Habbaa A., El-bagoury G. & Radwan M.E, 2011, ‘Isolation and identification of lumpy skin disease virus from naturally infected buffaloes at Kaluobia, Egypt’, *Global Veterinaria* 7, 234–237.

[CIT0018] El-Tholoth M. & El-Kenawy A, 2016, ‘G-protein-coupled chemokine receptor gene in lumpy skin disease virus isolates from cattle and water buffalo (Bubalus bubalis) in Egypt’, *Transboundary and Emerging Diseases* 63, e288–e295. https://doi.org/10.1111/tbed.12344 25754131 10.1111/tbed.12344

[CIT0019] Fagbo S., Coetzer J.A. & Venter E.H, 2014, ‘Seroprevalence of rift valley fever and lumpy skin disease in African buffalo (Syncerus caffer) in the Kruger National Park and Hluhluwe-iMfolozi Park, South Africa’, *Journal of the South African Veterinary Association* 85, 1075. 10.4102/jsava.v85i1.1075 25686252

[CIT0020] Gari G., Waret-Szkuta A., Grosbois V., Jacquiet P. & Roger F, 2010, ‘Risk factors associated with observed clinical lumpy skin disease in Ethiopia’, *Epidemiology and Infection* 138, 1657–1666. https://doi.org/10.1017/S0950268810000506 20233495 10.1017/S0950268810000506

[CIT0021] Greta A., Gourreau J.M., Vassart M., Wyers M. & Lefevre P.C, 1992, ‘Capripoxvirus disease in an Arabian oryx (Oryx leucoryx) from Saudi Arabia’, *Journal of Wildlife Diseases* 28, 295–300. https://doi.org/10.7589/0090-3558-28.2.295 1602585 10.7589/0090-3558-28.2.295

[CIT0022] House J.A., Wilson T.M., El Nakashly S., Karim I.A., Ismail I., El Danaf N. et al., 1990, ‘The isolation of lumpy skin disease virus and bovine herpesvirus-from cattle in Egypt’, *Journal of Veterinary Diagnostic Investigation* 2, 111–115. https://doi.org/10.1177/104063879000200205 1965577 10.1177/104063879000200205

[CIT0023] Hunter P. & Wallace D, 2001, ‘Lumpy skin disease in southern Africa: A review of the disease and aspects of control’, *Journal of the South African Veterinary Association* 72, 68–71. https://doi.org/10.4102/jsava.v72i2.619 11513262 10.4102/jsava.v72i2.619

[CIT0024] Kumar S.M, 2011, ‘An outbreak of lumpy skin disease in a Holstein dairy herd in Oman: A clinical report’, *Asian Journal of Animal and Veterinary Advances* 6, 851–859. https://doi.org/10.3923/ajava.2011.851.859

[CIT0025] Lubinga J.C., Clift S.J., Tuppurainen E.S., Stoltsz W.H., Babiuk S., Coetzer J.A. et al., 2014, ‘Demonstration of lumpy skin disease virus infection in *Amblyomma hebraeum* and *Rhipicephalus appendiculatus* ticks using immunohistochemistry’, *Ticks and Tick-borne Diseases* 5, 113–120. https://doi.org/10.1016/j.ttbdis.2013.09.010 24287140 10.1016/j.ttbdis.2013.09.010

[CIT0026] Magori-Cohen R., Louzoun Y., Herziger Y., Oron E., Arazi A., Tuppurainen E. et al., 2012, ‘Mathematical modelling and evaluation of the different routes of transmission of lumpy skin disease virus’, *Veterinary research* 43, 1 https://doi.org/10.1186/1297-9716-43-1 22236452 10.1186/1297-9716-43-1 PMC3268087

[CIT0027] OIE, 2010, *Terrestrial manual of lumpy skin disease*, Version adopted by the World Assembly of Delegates of the OIE in May 2010, OIE, Paris.

[CIT0028] Rweyemamu M., Paskin R., Benkirane A., Martin V., Roeder P. & Wojciechowski K, 2000, ‘Emerging diseases of Africa and the Middle East’, *Annals of the New York Academy of Sciences* 916, 61–70. https://doi.org/10.1111/j.1749-6632.2000.tb05275.x 11193683 10.1111/j.1749-6632.2000.tb05275.x

[CIT0029] Sharawi S. & El-Rahim I.A, 2014, ‘The utility of polymerase chain reaction for diagnosis of lumpy skin disease in cattle and water buffaloes in Egypt’, *Revue Scientifique et Technique-OIE* 30, 821 https://doi.org/10.20506/rst.30.3.2075 10.20506/rst.30.3.2075 22435194

[CIT0030] Shen Y.-J., Shephard E., Douglass N., Johnston N., Adams C., Williamson C. et al., 2011, ‘A novel candidate HIV vaccine vector based on the replication deficient Capripoxvirus’, Lumpy skin disease virus (LSDV), *Virology Journal* 8, 265 https://doi.org/10.1186/1743-422X-8-265 21624130 10.1186/1743-422X-8-265 PMC3117847

[CIT0031] Stram Y., Kuznetzova L., Friedgut O., Gelman B., Yadin H. & Rubinstein-Guini M, 2008, ‘The use of lumpy skin disease virus genome termini for detection and phylogenetic analysis’, *Journal of Virological Methods* 151, 225–229. https://doi.org/10.1016/j.jviromet.2008.05.003 18582954 10.1016/j.jviromet.2008.05.003

[CIT0032] Tageldin M.H., Wallace D.B., Gerdes G.H., Putterill J.F., Greyling R.R., Phosiwa M.N. et al., 2014, ‘Lumpy skin disease of cattle: An emerging problem in the Sultanate of Oman’, *Tropical Animal Health and Production* 46, 241–246. https://doi.org/10.1007/s11250-013-0483-3 24097247 10.1007/s11250-013-0483-3 PMC3895213

[CIT0033] Troyo A., Calderón-Arguedas O., Fuller D.O., Solano M.E., Avendaño A., Arheart K.L. et al., 2008, ‘Seasonal profiles of *Aedes aegypti* (Diptera: Culicidae) larval habitats in an urban area of Costa Rica with a history of mosquito control’, *Journal of Vector Ecology: Journal of the Society for Vector Ecology* 33, 76 https://doi.org/10.3376/1081-1710(2008)33[76:SPOAAD]2.0.CO;2 18697310 10.3376/1081-1710(2008)33[76:spoaad]2.0.co;2 PMC2560178

[CIT0034] Tuppurainen E. & Oura C, 2012, ‘Review: Lumpy skin disease: An emerging threat to Europe, the Middle East and Asia’, *Transboundary and Emerging Diseases* 59, 40–48. https://doi.org/10.1111/j.1865-1682.2011.01242.x 21749675 10.1111/j.1865-1682.2011.01242.x

[CIT0035] Tuppurainen E.S., Stoltsz W.H., Troskie M., Wallace D.B., Oura C., Mellor P.S. et al., 2011, ‘A potential role for ixodid (hard) tick vectors in the transmission of lumpy skin disease virus in cattle’, *Transboundary and Emerging Diseases* 58, 93–104. https://doi.org/10.1111/j.1865-1682.2010.01184.x 21114790 10.1111/j.1865-1682.2010.01184.x

[CIT0036] Tuppurainen E.S., Venter E. & Coetzer J, 2005, ‘The detection of lumpy skin disease virus in samples of experimentally infected cattle using different diagnostic techniques’, *Onderstepoort Journal of Veterinary Research* 72, 153–164. https://doi.org/10.4102/ojvr.v72i2.213 16137133 10.4102/ojvr.v72i2.213

[CIT0037] Tuppurainen E.S., Venter E.H., Coetzer J.A. & Bell-Sakyi L, 2015, ‘Lumpy skin disease: Attempted propagation in tick cell lines and presence of viral DNA in field ticks collected from naturally-infected cattle’, *Ticks and Tick-borne Diseases* 6, 134–140. https://doi.org/10.1016/j.ttbdis.2014.11.002 25468765 10.1016/j.ttbdis.2014.11.002 PMC4329317

[CIT0038] Weiss K, 1968, *Lumpy skin disease virus*, Cytomegaloviruses. Rinderpest Virus, Lumpy Skin Disease Virus, Springer, Berlin, pp. 111–131.

